# Single-Nucleotide Polymorphisms, *PITX2* and Abnormal Electrical Activity in Atrial Fibrillation

**DOI:** 10.3390/ijms26199780

**Published:** 2025-10-08

**Authors:** Verónica Jiménez-Sábado, Leif Hove-Madsen

**Affiliations:** 1 CIBER de Enfermedades Cardiovasculares, Instituto de Salud Carlos III, 28029 Madrid, Spain; vjimenezs@santpau.cat; 2 Institut de Recerca Sant Pau (IR SANT PAU), 08041 Barcelona, Spain; 3 Instituto de Investigaciones Biomédicas de Barcelona-Consejo Superior de Investigaciones Científicas (IIBB-CSIC), 08036 Barcelona, Spain

**Keywords:** Atrial arrhythmogenesis, PITX2 signaling pathways, genetic regulation, 4q25 risk variants, personalized medicine, ablation- and pharmacotherapy, cardiomyocyte function, calcium homeostasis, electrophysiology

## Abstract

Since single-nucleotide polymorphisms (SNPs) associated with increased risk of atrial fibrillation (AF) on chromosome 4q25 are located near the transcription factor PITX2, research has investigated relationships between SNPs, PITX2 activity and atrial function to improve risk stratification and identify new therapies. Although *PITX2* levels are heterogeneous, most studies converge towards lower *PITX2* levels in patients with AF, and a 4q25 SNP has been reported to reduce *PITX2* expression. However, there are several SNPs at 4q25 that segregate independently, and patients carrying different SNPs respond differently to ablation therapy. On the other hand, atrial-specific deletion of *Pitx2c* mimics molecular and electrophysiological alterations observed in patients with AF. This includes microRNAs, signaling pathways, ion channels, calcium homeostasis, electrical remodeling, contraction and the response to pharmacological treatments. Moreover, mutations in the *PITX2* homeodomain are associated with AF, PITX2 dysfunction or impaired calcium homeostasis. Interestingly, myocytes with the 4q25 risk allele rs13143308T display electrophysiological alterations similar to those reported in patients with AF or mice with heterozygous *Pitx2c* deletion. Moreover, carriers of rs13143308T respond poorly to ablation or antiarrhythmic drug therapy. Future research needs to establish how different 4q25 SNPs impact different *PITX2* isoforms and the downstream regulation of atrial function.

## 1. Introduction

Atrial fibrillation (AF) stands as the most prevalent sustained cardiac arrhythmia globally. Its prevalence is about 1–3% in the general population, but rises progressively with age, reaching around 9% in individuals over 65 and 17% by the age of 80 [1]. This irregular and rapid heart rhythm is a major public health concern, contributing to substantial morbidity and mortality, including a 2-fold increased risk of mortality [2] and a 4- to 5-fold increased risk of stroke [3]. While traditional risk factors such as advanced age, hypertension, obesity, and other cardiovascular diseases are well-established contributors [4,5,6], recent evidence highlights a strong genetic component. In certain populations, genetic factors may account for over 60% of the variance in AF susceptibility [7].

The emergence of Genome-Wide Association Studies (GWAS) has changed our understanding of the genetic architecture underlying complex diseases like AF. The first GWAS linking single-nucleotide polymorphisms (SNPs) to AF was published in 2007, identifying the SNPs rs2200733, rs10033464 and rs13143308 located on chromosome 4 in the intergenic region 4q25 [8]. This region has consistently been validated as the most robust AF risk SNP across diverse populations [8,9,10].

The gene closest to these 4q25 risk SNPs is the paired-like homeodomain transcription factor 2 (*PITX2*), which encodes the transcription factor PITX2 that plays a key role in cardiac development and the establishment of left–right asymmetry in the heart [11,12,13]. Moreover, *PITX2* had already been shown to regulate the expression of many proteins playing a key role in the regulation of atrial electrical activity such as ion channels, calcium-handling proteins, and intercalated disk components, many of which exhibit altered expression or activity in AF [14,15,16,17,18,19].

**Figure 1:**
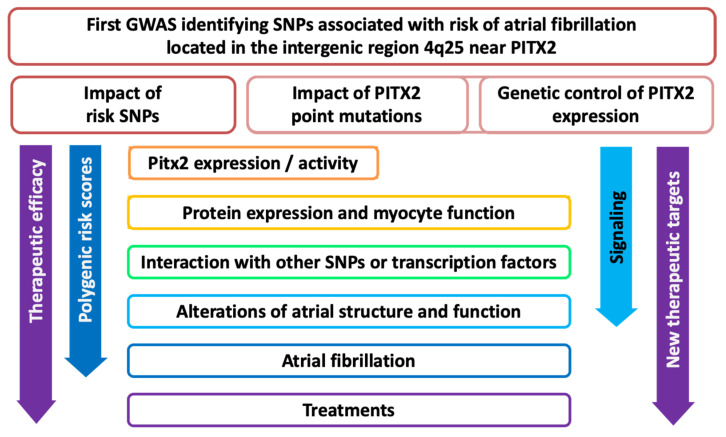
Schematic overview of the cascade of research emerging from the discovery of SNPs associated with increased risk of AF in the chromosomic region 4q25 near *PITX2*.

These findings have spurred new lines of research as outlined in Figure 1. Current research explores three major lines: (i) the impact of 4q25 risk SNPs on *PITX2* expression and activity, (ii) the effects of *PITX2* point mutations, and (iii) the genetic and molecular control of *PITX2* expression. The genetic variants converge on mechanisms regulating *PITX2* expression or activity, downstream protein expression and myocyte function, as well as interactions with other SNPs and transcription factors. This cascade leads to structural and functional atrial remodeling, culminating in atrial cardiomyopathy and AF and influencing therapeutic outcomes. The figure also highlights how insights into the impact of the cascade on polygenic risk scores, therapeutic efficacy, signaling pathways, and the identification of novel therapeutic targets are being integrated into efforts to personalize the treatment of AF.

## 2. Single-Nucleotide Polymorphisms Affecting Atrial Electrical Activity

### 2.1. Genome-Wide Association Studies

The landmark study by Gudbjartsson et al. in 2007 [8] was the first to identify two sequence variants on chromosome 4q25, rs2200733 and rs10033464, associated with a significant increase in the risk of AF. For instance, the rs2200733T allele alone conferred an odds ratio of 1.90 in combined analyses, highlighting its substantial contribution to AF susceptibility [20]. Subsequent larger meta-analyses of GWAS have significantly expanded the number of AF-associated loci, identifying over 350 genomic regions implicated in AF [9,21,22,23,24], although the 4q25 region consistently shows the strongest association. The strength of this association at 4q25 has been replicated across multiple ancestries, including European, East Asian, and African populations [8,9,10,20,23], underscoring its robust predictive power.

However, despite the strong statistical associations, the precise functional consequences of many non-coding SNPs identified by GWAS remain elusive. A particular challenge arises because these SNPs are often located in intergenic regions, far from the nearest gene, as is the case for 4q25 SNPs and *PITX2*. The challenge lies in determining which are the true causal SNPs, as SNPs may simply be in strong linkage disequilibrium with actual causal variants residing within regulatory regions [25].

### 2.2. Impact of Risk SNPs on PITX2 Expression or Function

Since most 4q25 risk SNPs are located in non-coding regions, positioned approximately 150–171 kb upstream of the *PITX2* gene, initial hypotheses suggested that these variants might alter *PITX2* expression. However, determining how AF-associated SNPs influence *PITX2* expression has proven challenging, and subsequent studies have reported inconsistent findings. Three isoforms of *PITX2* have been identified in humans: *PITX2a*, *PITX2b* and *PITX2c*. *PITX2a* and *PITX2b* are produced from a common promoter, whereas *PITX2c* is the product of an alternative promoter and is the main isoform expressed in the heart [17]. One study in 239 human left atrial samples found no influence of risk SNPs from four different linkage groups (rs1448818, rs2200733, rs10033464 or rs3853445) on *PITX2c* mRNA levels [25]. Supporting this notion, another study reported no effect of the risk alleles at rs1448818, rs2200733 or rs6838973 on *PITX2c* mRNA expression levels [19]. On the other hand, a separate investigation revealed that total *PITX2a* expression was doubled in the presence of risk alleles at rs17042171 (in the same linkage group as rs2200733) or rs6843082 (linked with rs10033464), while these variants did not influence *PITX2c* levels [26]. In addition, more refined approaches focusing on cardiomyocyte-specific expression have demonstrated that reduced left atrial cardiomyocyte *PITX2* concentrations, but not whole-tissue *PITX2* levels, are associated with AF recurrence after ablation [27]. This highlights the importance of cell-type-specific analysis when studying *PITX2* function.

One proposed mechanism by which 4q25 risk SNPs influence *PITX2* expression is their location within enhancer regions that are known to possess long-range transcriptional regulatory function. In line with this, a study identified long-range regulatory interactions between the 4q25 region and the *PITX2* promoter through chromosome conformation capture studies [28]. Another study found that the SNP rs2595104 (in the same linkage group as rs1448818) reduced *PITX2c* expression through interaction with the enhancer binding protein TFAP2a [29]. These findings suggest that AF-associated variants may act through complex regulatory mechanisms involving chromatin looping and three-dimensional genome organization rather than simple linear distance effects.

The conflicting results across studies may also indicate that the effect of SNPs is context-dependent, potentially exerting greater influence during key stages of cardiac development or within specific atrial regions where *PITX2* expression is particularly high [25], as reported in the pulmonary vein region of 3-day-old mice [30]. Another possibility is that epigenetic alterations in the adult diseased atrium may conceal the underlying genetic effects of a risk SNP. These observations underscore the complexity of translating GWAS-identified associations into detailed molecular insights in adult tissues. They also emphasize the importance of conducting spatio-temporal analyses of gene expression and epigenetic modifications in distinct atrial cell types and developmental stages, in order to clarify how non-coding SNPs influence *PITX2* function and contribute to AF pathogenesis.

### 2.3. Impact of Risk SNPs on Cardiomyocyte Function

Although numerous SNPs have been associated with AF, their precise functional effects remain largely undefined. However, a growing body of functional evidence supports the hypothesis that AF-associated SNPs at chromosome 4q25 have direct effects on cardiomyocyte electrophysiology and calcium handling, even in the absence of diagnosed AF or altered *PITX2* expression. These effects likely contribute to AF susceptibility by creating a proarrhythmic substrate at the level of the atrial myocyte. One of the most well-characterized risk variants is rs13143308T, and a pivotal study by Herraiz-Martínez et al. demonstrated for the first time that right atrial cardiomyocytes from patients without a history of AF that carried this variant exhibited electrophysiological remodeling similar to that observed in patients with AF [31]. Specifically, atrial myocytes from patients carrying rs13143308T allele(s) showed a significantly higher incidence of calcium waves, transient inward currents (I_TI_), and spontaneous membrane depolarizations. These electrophysiological alterations were mechanistically linked to higher sarcoplasmic reticulum (SR) calcium loading, increased sarcoplasmic reticulum Ca^2+^-ATPase 2a (SERCA2a) expression, and enhanced ryanodine receptor 2 (RyR2) phosphorylation at serine 2808.

Despite these insights, functional modeling of non-coding AF risk SNPs remains limited at this point, primarily due to technical challenges in precisely editing non-coding loci in relevant human cell or animal models.

### 2.4. Impact of Risk SNPs on Atrial Function

The influence of 4q25 risk variants extends beyond cellular electrophysiology to atrial tissue and atrial myocardial functions such as conduction velocity, mechanical contraction, and structural remodeling susceptibility. Thus, clinical studies have demonstrated that individuals carrying these 4q25 SNPs often present a more arrhythmogenic atrial substrate, even during sinus rhythm [32,33,34]. For instance, Husser et al. reported that individuals with the rs2200733 risk allele exhibited a shorter atrial effective refractory period and increased susceptibility to complex arrhythmias during electrophysiological assessment [32], indicating a genetically driven atrial dysfunction that may precede clinically apparent AF. From a mechanical perspective, the rs10033464 risk SNP has been associated with increased left atrial volume [30], a change that can compromise atrial compliance and contractility. These mechanical alterations may in turn promote electrical and structural remodeling, fostering a proarrhythmic substrate that contributes to AF susceptibility. Another study associated the rs2200733 variant with prolonged PR interval in patients with and without AF [34].

### 2.5. Impact of Risk SNPs on AF Therapy

The clinical relevance of 4q25 variants also extends beyond disease risk, influencing therapeutic outcomes in AF. Indeed, individuals carrying AF-associated variants in the 4q25 region exhibit altered responses to specific treatments, with multiple studies reporting that carriers of rs2200733 or rs10033464 risk variants [32,35,36,37,38,39], or other SNPs from one of these two linkage groups [40], have significantly higher rates of AF recurrence following catheter ablation. In line with this, the rs2200733 and rs17570669 risk alleles have been identified as independent predictors of AF recurrence after successful cardioversion [41,42].

On the other hand, a 4q25 SNP that increases P-wave duration has been reported to decrease AF risk [43]. The efficacy of antiarrhythmic drug therapy also appears to be modulated by the 4q25 genotype. One study found that patients with the normal allele at rs10033464 responded more favorably to class III antiarrhythmic drugs, whereas those with the risk allele had a better response to class I agents [44]. Table 1 summarizes the reported impact of AF-associated risk SNPs at 4q25 on *PITX2* expression, atrial function, and the implications for risk stratification and therapy.

Together, these findings lend support to the potential utility of AF-associated risk SNPs as an additional tool in precision medicine-based management of AF, where integration of genetic profiling in combination with biomarker analysis could help guide clinical decisions on patient selection for catheter ablation and antiarrhythmic drug therapy. However, clinical translation is currently limited by modest effect sizes of single SNPs, and findings are not always consistent across studies. Therefore, polygenic mechanism-based risk scores and large-scale prospective validation studies are necessary to advance towards the implementation of SNPs as tools in clinical practice.

## 3. Modulation of Electrical Activity by PITX2

### 3.1. Relationship Between PITX2 and AF

The relationship between *PITX2* and AF susceptibility has been extensively studied in both human patients and animal models. Most studies in human atrial samples demonstrate that reduced *PITX2* expression is associated with AF [16,17]. Analyses of left atrial tissue from AF patients typically showed lower *PITX2* mRNA and protein levels compared to those in controls with sinus rhythm. *PITX2* deficiency has been proposed to contribute to the arrhythmogenic substrate by altering the transcriptional regulation of key ion channels and calcium-handling proteins. Several studies reported alterations in TASK-2, K_v_7.1, HCN4, Na_v_1.5, Kir2.1, K_v_1.3, K_v_3.4 and Ca_v_1.2, which may influence action potential duration (APD) and resting membrane potential [16,17,19,30], thereby contributing to a proarrhythmic substrate that facilitates AF initiation. Regarding the alterations in calcium-handling proteins, *PITX2* deficiency has been linked to an increase in *Atp2a2* (SERCA2a) [15,18], *Casq2* (CASQ2) [15] and *Pln* (PLN) [15]. These changes were associated with altered SR calcium uptake and release [15], which are key hallmarks of AF pathophysiology.

Conversely, another study suggested that not only a reduction but also an excess of *PITX2* may favor AF. In a subset of AF patients, miRNA profiling and transcriptomic studies have revealed abnormally high *PITX2* expression associated with electrical remodeling. This remodeling is characterized by a shortened APD, driven by decreased L-type calcium current (I_CaL_) and increased slow rectifier potassium current (I_Ks_) [45], changes that enhance reentrant activity and contribute to a proarrhythmic atrial substrate.

Together, these findings suggest that the relationship between *PITX2* expression and AF risk follows a biphasic pattern, in which both overexpression and deficiency may promote arrhythmogenesis by distinct mechanisms. Reduced *PITX2* disrupts transcriptional control of ion channels and calcium-handling proteins, leading to slowed conduction, impaired SR calcium cycling, and structural remodeling that together create a substrate vulnerable to AF. Excessive *PITX2*, in contrast, promotes electrical remodeling through shortened APD and increased susceptibility to reentry. This suggests that *PITX2* must be tightly balanced, as both loss and excess may destabilize atrial electrophysiology. Moreover, the results obtained from animal models frequently demonstrate clearer causal links between *PITX2* perturbation and arrhythmia phenotypes, but these systems differ from human disease in developmental timing, genetic background and the absence of human comorbidities. Thus, heterogeneous findings across experimental and clinical studies can be reconciled when different factors are taken into account: (i) the tissue used for the assay (whole tissue vs. isolated cardiomyocytes or single-cell approaches), (ii) the isoform(s) analyzed (*PITX2a*, *PITX2b* or *PITX2c*), (iii) the timing of measurement (developmental, early disease vs. advanced/remodeled atrium), and (iv) interspecies differences and environmental modifiers (comorbidities, medications, epigenetic changes). These variables can mask or invert an underlying genetic effect and should be explicitly considered when interpreting outcomes. From a translational perspective, these insights imply that therapeutic strategies should focus not on simply increasing or suppressing *PITX2*, but rather on restoring and maintaining physiological expression levels to preserve normal atrial function.

### 3.2. PITX2 Point Mutations and Non-Coding RNA

Several point mutations in the *PITX2* gene have been identified in patients with AF [46,47,48,49,50]. These mutations typically occur in the homeodomain region and result in loss of function or altered DNA binding capacity. For instance, in one study, the *PITX2c* mutations MD4 and MD5 were shown to impair the transcriptional activity of the protein, as demonstrated by reduced Nppa-luciferase transactivation and diminished repression of the Shox2 promoter [46]. When expressed in cardiomyocytes, these mutant variants disrupt normal expression of calcium-handling proteins and alter the SR calcium loading. This leads to irregular calcium transient amplitudes and deterioration of beat-to-beat stability, thereby establishing a direct link between *PITX2* coding mutations and impaired cellular electrophysiological function.

However, a study also identified a *PITX2c* gain-of-function mutation in patients with AF [51]. To investigate its functional consequences in human cardiomyocytes, human induced pluripotent stem cell (hiPSC) lines derived from a patient with AF carrying the heterozygous mutation and its isogenic control obtained by CRISPR/Cas9 correction were generated [52]. Building on earlier evidence showing that *PITX2c* regulates oxidative phosphorylation and redox balance in murine atrial cardiomyocytes [53], Benzoni et al. [54] employed these iPSC-derived models to assess the impact of the mutation on mitochondrial function. Their findings revealed that the *PITX2c* mutation leads to enhanced oxidative respiration, indicating mitochondrial hyperactivity. This mitochondrial overwork may contribute to arrhythmogenesis, as several studies have highlighted the role of dysregulated cytosolic-mitochondrial calcium coupling in promoting proarrhythmic conditions [55,56,57].

The regulation of *PITX2* by non-coding RNAs, particularly microRNAs (miRs) and long non-coding RNAs (lncRNAs), has become a key area of investigation since several studies reported their dysregulation in AF [58,59]. *PITX2* expression has been linked to changes in a broad array of miRNAs, including miR106b-25, miR-17-92, miR-21, miR-1, miR-26b, miR-29a, miR-30e, miR-106b, miR-133, miR-200a, miR-106a, miR-203, miR-208a and miR-208b [15,60,61]. Notably, one study demonstrated that *PITX2* positively regulates miR-17-92 and miR-106b-25. Deficiency of these miRs in mouse models led to pacing-induced AF, prolonged PR intervals, and sinus node dysfunction [60]. The same study further revealed that these miRNAs suppress genes essential for sinoatrial node development, such as *Shox2* and *Tbx3*, thereby providing the first genetic evidence linking microRNAs to AF pathogenesis [60]. Among the lncRNAs, the *Pitx2* adjacent non-coding RNA (PANCR) is expressed in the human left atrium and exhibits a strong positive correlation with *PITX2c* mRNA levels [62]. Silencing PANCR significantly reduces *PITX2c* expression, and the resulting transcriptomic changes closely resemble those observed following *Pitx2c* knockdown, highlighting PANCR as a crucial upstream regulator. Playrr and Fendrr, other lncRNAs, have been proposed to modulate *Pitx2* expression [63,64,65]. Playrr has been shown to repress *Pitx2* within the left–right asymmetry pathway by interfering with promoter activity [65]. On the other hand, in silico approaches suggest that Fendrr structure may facilitate a direct binding to *Pitx2* promoters through the formation of a DNA:RNA triplex structure, although it requires experimental validation [63,64]

The identification of *PITX2* point mutations, along with its regulation by non-coding RNAs, reveals that *PITX2* is controlled by a complex, multilayered network. This, points to therapeutic strategies for AF that extend beyond targeting the *PITX2* gene itself. Specifically, modulating certain non-coding RNAs or correcting the functional consequences of point mutations may offer novel and highly specific therapeutic approaches.

### 3.3. Genetic Manipulation of PITX2 and Experimental Models of Pitx2 Deficiency

**Figure 2:**
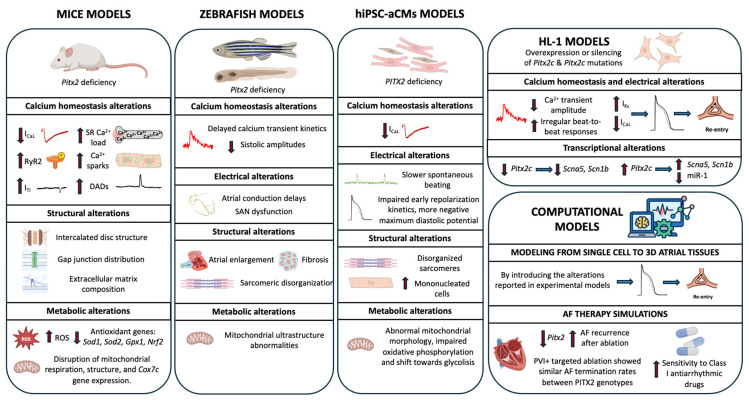
Overview of *PITX2* models and key findings. I_CaL_:L-type calcium current; RyR2: ryanodine receptor 2; I_TI_: transient inward current; SR: sarcoplasmic reticulum; DADs: delayed afterdepolarizations; ROS: reactive oxygen species; SAN: Sinoatrial node I_Ks_: Slow rectifier potassium current.

Studies to date have examined *PITX2* function across multiple systems, from animal models such as mice and zebrafish to hiPSC-aCMs, HL-1 cells, and computational platforms. Nevertheless, interspecies variation in electrophysiological properties presents a significant challenge for translational research. Parameters such as APD, ion channel expression, and cardiac structure differ markedly across species (e.g., rodents, rabbits, zebrafish and large mammals), influencing both susceptibility to AF and the response to therapeutic interventions. Crucially, many animal models primarily exhibit acute or inducible arrhythmias rather than the sustained, remodeling-driven forms observed in humans. Consequently, modeling the gradual electrophysiological and structural remodeling characteristic of human chronic AF remains a critical obstacle when using animal models, limiting the ability to fully recapitulate human disease pathophysiology and to evaluate long-term therapeutic strategies. Figure 2 summarizes different models used to date to investigate *PITX2* role, along with the principal findings associated with each.

#### 3.3.1. Mouse Models

Mouse models have been instrumental in establishing a causal role for *PITX2* in the molecular and electrophysiological alterations underlying AF susceptibility. Multiple Pitx2-deficient mouse models have consistently demonstrated electrophysiological and structural changes that promote AF vulnerability [15,16,17,30,66,67,68].

Atrial-specific deletion of *Pitx2*, using models such as NppaCrePitx2 mice, leads to dose-dependent dysregulation of calcium-handling mechanisms, including reduced I_CaL_ density and altered sarcoplasmic reticulum (SR) calcium load [15]. Heterozygous atrial-specific deletion of *Pitx2* resulted in increased phosphorylation of RyR2, spontaneous calcium release events, elevated incidence of I_TI_, and spontaneous electrical activity [14]. These phenotypes closely mimic alterations observed in atrial myocytes from patients carrying 4q25 risk variants [31] and from patients with AF [69,70,71,72,73,74,75,76].

Structural remodeling is another hallmark of *Pitx2* deficiency, characterized by alterations in intercalated disk structure, gap junction distribution, and extracellular matrix composition [15,16,17,18]. These changes disrupt atrial conduction and generate a substrate favorable for re-entrant arrhythmias. At the tissue level, *Pitx2*-deficient atria exhibit shortened APD, increased interatrial electrical heterogeneity, and heightened propensity for pacing-induced AF [17,30]. The left atrial-specific expression of *Pitx2* contributes to intrinsic electrical asymmetry, which may increase vulnerability to AF when disrupted.

Metabolic disturbances have also been observed in *PIitx2*-deficient mouse atria [66,67]. Thus, Subati et al. [66] revealed that *Pitx2* haploinsufficiency in mouse atria causes significant oxidative stress and mitochondrial dysfunction, characterized by increased reactive oxygen species (ROS), impaired mitochondrial respiration, and disrupted mitochondrial networks. This metabolic impairment includes downregulation of antioxidant genes (such as *Sod1*, *Sod2*, *Gpx1*, *Nrf2*), leading to redox imbalance and bioenergetic failure. Similarly, Li et al. reported accumulation of adipose-like tissue, markers of oxidative stress, mitochondrial structure abnormalities, and impaired expression of mitochondrial genes such as *Cox7c* in *Pitx2* conditional knockout mice [67]. Importantly, these metabolic derangements contribute to the development of a proarrhythmic substrate.

Complementary transcriptomic and epigenomic analyses by Steimle et al. [68] revealed that *Pitx2* disruption affects cardiomyocyte-specific gene regulatory networks critical for ion channel function, contractile function, and mitochondrial metabolism within both the pulmonary veins and left atrium in the Pitx2-deficient mouse model.

While mouse models have been essential in demonstrating causal links between *Pitx2* deficiency and AF susceptibility, their translational relevance is limited by important differences from human atrial biology. Mice have markedly shorter APDs, higher intrinsic heart rates, and distinct ion channel expression patterns compared to humans [77]. Moreover, genetic manipulations often result in more pronounced phenotypes than those observed in human carriers of common AF-associated variants, which typically exert modest effect sizes. Finally, most mouse studies are performed in animals without comorbidities, whereas human AF usually develops in the context of comorbidities such as hypertension [78], obesity [79], or sleep apnea [80,81,82]. These differences must be taken into account when extrapolating findings from mouse *Pitx2* models to clinical AF.

#### 3.3.2. Zebrafish Model

Zebrafish models have provided valuable insights into the developmental and electrophysiological consequences of *Pitx2c* deficiency [83,84]. In adult *Pitx2c*-null zebrafish, Collins et al. demonstrated atrial remodeling characterized by conduction delays, atrial enlargement, fibrosis, sarcomeric disorganization, and mitochondrial ultrastructural abnormalities. These structural and metabolic alterations were associated with spontaneous arrhythmic activity, and antioxidant treatment partially mitigated the phenotype, implicating oxidative stress as a contributing factor [84]. More recently, studies in *Pitx2c* mutant zebrafish larvae revealed sinoatrial node dysfunction and atrial-specific calcium-handling abnormalities, including reduced systolic amplitude and delayed calcium transient kinetics. These early defects in atrial excitation–contraction coupling may predispose to later arrhythmogenic remodeling [83].

Collectively, these models underscore the critical role of *Pitx2* in maintaining atrial electrical and metabolic homeostasis. However, the zebrafish heart is structurally simpler, with a single atrium and ventricle, and the electrophysiological properties differ substantially from mammals. Thus, while zebrafish models are powerful for dissecting pathways and developmental roles of *Pitx2*, caution is needed when extrapolating findings from zebrafish to human AF pathophysiology [85].

#### 3.3.3. Human Induced Pluripotent Stem Cell (hiPSC) Model

Human induced pluripotent stem cell (hiPSC) models have emerged as an important model for translational studies of molecular mechanisms contributing to the induction of AF in humans [54,86,87,88]. Specifically, CRISPR/Cas9-mediated deletion of *PITX2* in hiPSC-derived atrial cardiomyocytes (hiPSC-aCMs) have provided novel insights into the molecular and functional consequences of *PITX2* deficiency relevant to AF pathogenesis. Schulz et al. [86] reported that PITX2-deficient hiPSC-aCMs had reduced contractility and slower spontaneous beating compared to controls. At the ion channel level, they showed that these cells exhibited significant downregulation of *CACNA1C*, the gene encoding the α1C subunit of the L-type calcium channel, resulting in diminished L-type calcium current density. Functionally, this translated into impaired early repolarization kinetics and a more negative maximum diastolic potential, hallmarks of electrical remodeling observed in AF patients. These findings suggest that *PITX2* loss induces primary electrical defects independent of the secondary effects of rapid atrial pacing or other external stressors. Structurally, Reyat et al. [87] observed disorganized sarcomeres, increased mononucleated cells, and abnormal mitochondrial morphology in CRISPR-edited *PITX2*-deficient hiPSC-aCMs. Functionally, mitochondria showed impaired oxidative phosphorylation and a shift toward glycolysis- features linked to atrial myopathy and arrhythmogenesis. Moreover, Benzoni et al. [54] also observed alterations in mitochondrial activity in hiPSC-aCMs harboring a *PITX2* gain-of-function mutation.

#### 3.3.4. HL-1 Model

The HL-1 atrial cardiomyocyte cell line provides a versatile in vitro system to investigate the multifaceted role of *Pitx2* in atrial electrophysiology and transcriptional regulation relevant to AF. Multiple studies using HL-1 cells have elucidated how *Pitx2* modulates ion channel expression, calcium handling, transcriptional networks, and cellular homeostasis [16,45,46].

Chinchilla et al. [16] utilized HL-1 cells to demonstrate that *Pitx2* insufficiency leads to both electrical and structural remodeling. Their work showed that *Pitx2* indirectly modulates I_K1_ via miR-1, while directly regulating *Scna5* expression. These findings support the notion that *Pitx2* alterations disrupt ion channel expression, contributing to the formation of arrhythmogenic molecular substrates. On the other hand, Pérez-Hernández et al. [45] advanced this understanding by showing that *Pitx2c* directly increases the I_Ks_ current in HL-1 cells through transcriptional upregulation of *Kcnq1* and *Kcne1*, major components of atrial repolarization currents. Interestingly, they also reported that *Pitx2c* indirectly modulates I_CaL_ via atrial natriuretic peptide-mediated pathways, thereby orchestrating a broader electrophysiological remodeling that affects atrial APD and excitability. More recently, Herraiz-Martínez et al. [46] investigated different AF-associated mutations in *Pitx2* using HL-1 cells. Certain mutations were found to disrupt calcium homeostasis, evidenced by reduced calcium transient amplitude, increased beat-to-beat variability, decreased SR calcium load, and downregulation of *Serca2*, *Pln* and *Casq* transcripts.

#### 3.3.5. Computational Models

Computational modeling has become a powerful tool for elucidating how *PITX2* dysfunction contributes to atrial electrophysiology remodeling and increased AF susceptibility. Multi-scale simulations- ranging from single-cell ion channel dynamics to 3D atrial tissue models- consistently demonstrate that *PITX2*-related electrical (e.g., increased slow delayed rectifier potassium current (I_Ks_) and reduced I_CaL_) and structural (e.g., increased fibrosis or decreased cell–cell coupling) remodeling can lead to APD shortening and conduction disturbances. These alterations promote reentrant excitation mechanisms and AF maintenance [89,90].

Expanding these insights to the organ level, a three-dimensional human atrial model incorporating patient-specific anatomy and fiber orientation showed that *PITX2* haploinsufficiency (*PITX2*+/−) increases AF sustainability, characterized by higher dominant frequencies and more complex, stable re-entrant wave dynamics compared to wild-type conditions [91]. Syeda et al. [19] further demonstrated, using computational modeling, that *PITX2* deficiency depolarizes atrial resting membrane potential (RMP) by downregulating the potassium channel TASK-2.

Beyond mechanistic understanding, computational models have also been employed to evaluate how *PITX2* deficiency influences the efficacy of AF therapies, particularly catheter ablation and antiarrhythmic drug interventions [19,90,91,92,93]. Virtual ablation simulations suggest that pulmonary vein isolation, when combined with targeted ablation, yields comparable AF termination rates across different *PITX2* genotypes, indicating that ablation efficacy may be generally preserved despite genetic variation [91]. Interestingly, several studies have shown increased sensitivity to class I antiarrhythmic drugs in *PITX2*-deficient models [19,90,92,93]. Mechanistically, this is attributed to *PITX2*-related RMP depolarization via TASK-2, which enhances post-repolarization refractoriness and thereby potentiates the effect of sodium channel blockers [19,94]. However, another study suggests that the antiarrhythmic efficacy of flecainide in the context of impaired PITX2 cannot be fully explained by its sodium-channel blocking properties. Instead, flecainide appears to exert additional benefits by suppressing spontaneous calcium release and increasing the wavelength of reentrant circuits [90].

By integrating molecular, cellular and tissue-scale data, these computational frameworks offer valuable mechanistic insights into how *PITX2* alterations contribute to AF substrates and variable drug responses. Incorporating patient-specific anatomical and fibrosis profiles further enables personalized simulation of AF dynamics, supporting individualized therapeutic strategies, including virtual ablation planning and in silico drug testing [95].

Calcium-handling, electrophysiological, structural and metabolic alterations observed across the different experimental models and their alignment with alterations observed in AF patients are summarized in Table 2.

### 3.4. Relationship Between PITX2 and AF Therapy

The expression level of *PITX2* in left atrial cardiomyocytes plays a significant role in determining the success of AF therapy, encompassing both catheter ablation and antiarrhythmic drug treatment. In a pivotal clinical study, Reyat et al. (2020) [27] found that reduced *PITX2* mRNA concentration in left atrial cardiomyocytes, but not in whole atrial tissue, was associated with a higher risk of AF recurrence following catheter ablation. Notably, each unit increase in *PITX2* expression was linked to a 16% reduction in recurrence risk. In the same cohort, BMP10, a *PITX2*-repressed plasma protein, emerged as an independent predictor of AF recurrence post-ablation. These findings support BMP10’s potential as a blood-based biomarker for risk stratification in AF management. Despite these genetic associations, recent computational modeling found that the efficacy of pulmonary vein isolation (PVI) ablation is preserved across *PITX2* genotypes [91].

The predictive strength of *PITX2* expression levels for recurrence appears modest compared to studies evaluating the impact of 4q25 on AF recurrence [32,35,36]. For example, Husser et al. (2010) [32], reported an increase in AF recurrence from 8% to 25% in patients carrying any rs2200733 or rs10033464 4q25 risk variants. Shoemaker et al. (2013) [35] found that individuals with a risk allele in rs2200733 had a 30% increased risk of atrial tachyarrhythmia recurrence. Similarly, He et al. (2016) [36] observed AF recurrence risks of 48% (rs2200733), or 51% (rs10033464) in carriers of at least one risk allele.

By contrast, antiarrhythmic drug response appears more sensitive to *PITX2* genotype. In particular, Class I sodium channel blockers demonstrated enhanced rhythm control in *PITX2* experimental and computational studies [19,90,94], making *PITX2* a promising biomarker for guiding pharmacological rhythm-control strategies.

Nonetheless, the predictive power of *PITX2* expression and related biomarkers remains modest, and their role in clinical decision-making is not yet standardized. Most of the supporting evidence comes from retrospective studies with limited cohort sizes, and multi-center prospective validation is still lacking. Furthermore, differences between whole-tissue and cell-specific measurements, as well as variability in isoform expression, complicate interpretation. As such, *PITX2*-guided therapy should currently be considered investigational, with future trials required to determine its utility in clinical practice.

## 4. Cross-Talk Between PITX2 Signaling, Risk SNPs and Other Transcription Factors

### 4.1. PITX2 and 4q25

Accumulating evidence suggests that the relationship between *PITX2* and the broader 4q25 region is complex, involving multiple cis-regulatory elements, chromatin architectural features, and other potential target genes that collectively influence AF susceptibility.

Recent integrative genomic analyses have mapped the three-dimensional chromatin interactions within the 4q25 AF-associated region, revealing multiple cis-regulatory elements (enhancers) located hundreds of kilobases away that physically contact the *PITX2* promoter through chromatin looping [68]. These enhancer–promoter interactions are key regulators of *PITX2* transcription in atrial cardiomyocytes, modulating gene expression in a tissue- and developmental stage-specific manner. Functional studies using CRISPR/Cas9-mediated deletions and reporter assays have identified specific enhancer elements that drive *PITX2* expression, abnormal atrial electrophysiology, and enhanced vulnerability to AF [28,98], providing direct mechanistic evidence that these regulatory elements contribute causally to AF pathogenesis through *PITX2* dysregulation. Beyond *PITX2*, the 4q25 variants also influence the expression of other genes and regulatory elements that may independently or synergistically contribute to AF susceptibility. Notably, *ENPEP*, a gene adjacent to *PITX2*, is regulated by some of the same enhancer elements within 4q25 [25,28]. *ENPEP* encodes glutamyl aminopeptidase, involved in the renin–angiotensin system and blood pressure regulation, suggesting that variants affecting *ENPEP* expression may influence cardiac electrophysiology and AF risk through distinct pathways from *PITX2*. On the other hand, another study demonstrated that AF-associated alleles can disrupt transcription factor binding, such as *TFAP2a* binding to rs2595104, resulting in reduced *PITX2c* transcription [29]. These results suggest that 4q25 risk SNPs may influence AF risk through effects on multiple genes rather than *PITX2* alone.

### 4.2. PITX2 and Other Transcription Factors

*PITX2* is a central transcriptional regulator that governs the expression of numerous genes essential for atrial development, electrical stability, and cardiomyocyte homeostasis. Its regulatory activity is integrated within a broader network of transcription factors, including members of the T-box, SMAD, and Forkhead families [68,99,100,101].

One of the most influential regulatory circuits involving *PITX2* is the Wnt signaling pathway, where *PITX2* functions both as a downstream effector and an upstream modulator. This bidirectional relationship forms a feedback loop that shapes cardiogenesis and atrial electrophysiology [15,102]. *TBX5*, another essential cardiac transcription factor, directly activates *PITX2*. Interestingly, *TBX5* and *PITX2* exhibit antagonistic regulatory effects on membrane effector genes, including *SCN5A* (sodium channel), *GJA1* (connexin 43), *RYR2* (RyR2), *DSP* (desmoplakin), and *ATP2A2* (SERCA2a), highlighting the importance of balanced transcriptional inputs in maintaining electrophysiological integrity [101]. Functionally, *PITX2* also cooperates with *NKX2.5*, a critical determinant of pulmonary myocardium identity, a region frequently implicated as a trigger site for AF [103]. Mutations in *PITX2* can disrupt this synergy, abolishing the cooperative transcriptional activation with *NKX2.5* and other regulators such as *GATA4*, thereby compounding the risk of transcriptional dysregulation and arrhythmogenesis [104]. Emerging evidence also points to transcriptional crosstalk between *Pitx2* and *Nr4a3* [68], which encodes the Neuron-derived Orphan Receptor-1 (NOR-1). Notably, NOR-1 expression has been reported to be dysregulated in atrial tissue from patients with AF [105,106].

Beyond its roles in development and electrophysiology, *PITX2* also modulates cardiomyocyte oxidative stress responses through direct interaction with *NRF2*, a master regulator of redox homeostasis [53]. This expands *PITX2*’s functional scope to include metabolic protection, which may be especially relevant in AF-associated structural and energetic remodeling.

This network-oriented perspective emphasizes the importance of studying how perturbations in a single transcription factor can propagate across multiple pathways, affecting atrial structure, electrophysiology, and arrhythmia susceptibility. Multi-omics approaches, including transcriptomics, epigenomics and proteomics, combined with computational network modeling, offer powerful strategies to map these interactions, identify critical network nodes, and predict consequences of dysregulation. Such integrative system biology models have the potential to reveal novel therapeutic targets and shift the focus from single-gene interventions towards network-informed strategies for AF treatment.

### 4.3. Pitx2 and Other SNPs

Although no SNPs outside the 4q25 region have been definitively linked to *PITX2* expression in adult human tissue, recent integrative genomics studies suggest that many AF-associated genetic variants converge functionally on *PITX2*-regulated pathways. Multiomic analyses, including transcriptomics, epigenomics, and proteomics, have revealed that the broader *PITX2* transcriptional network is significantly enriched for genes associated with AF [18,68,87,107]. These findings support a model in which *PITX2* serves as a central regulatory node within an extended architecture influenced by multiple genetic loci.

While the 4q25 region remains the most robust and reproducible genetic locus linked to AF, particularly due to its proximity to *PITX2*, emerging evidence indicates that SNPs at other loci may modulate components of the same transcriptional, electrophysiological, or structural pathways. For example, loci near *ZFHX3* [108,109], *KCNN3* [110,111,112], *PRRX1* [113], and *CAV1* [114,115] have been implicated in AF risk. Several of these genes are either directly regulated by or functionally interact with *PITX2*-dependent processes [15]. Moreover, network-based analyses of GWAS data have identified gene modules enriched for AF heritability that are transcriptionally connected to *PITX2* activity, underscoring the potential for epistatic interactions or pathway-level convergence [116].

Taken together, while 4q25 remains the key genomic hotspot for *PITX2*-mediated susceptibility, a more nuanced picture is emerging in which polygenic risk variants across the genome may directly or indirectly influence *PITX2* activity or amplify its downstream effects. These insights underscore the value of systems-level approaches in deciphering the complex genetic architecture of AF, shifting the focus from isolated loci toward integrated regulatory networks.

## 5. Future Directions and Limitations

Since Gudbjartson et al. [8] demonstrated the association of 4q25 SNPs and AF- implicating *PITX2* as a key molecular effector, research outlined in this review has revealed complex regulatory networks that modulate *PITX2* expression and function. However, future lines of research are required to elucidate isoform-specific, spatiotemporal, and context-dependent regulation of *PITX2*.

A central priority will be to clarify how lead SNPs from distinct linkage groups within the 4q25 region modulate the expression and function of *PITX2* isoforms (*PITX2a*, *PITX2b*, *PITX2c*) across cardiac tissues, cell types, and developmental stages. In particular, understanding how these regulatory mechanisms differ between the atria and pulmonary veins will be crucial. This effort will require single-cell and spatial transcriptomics, long-read sequencing, and other high-resolution omics approaches.

**Figure 3:**
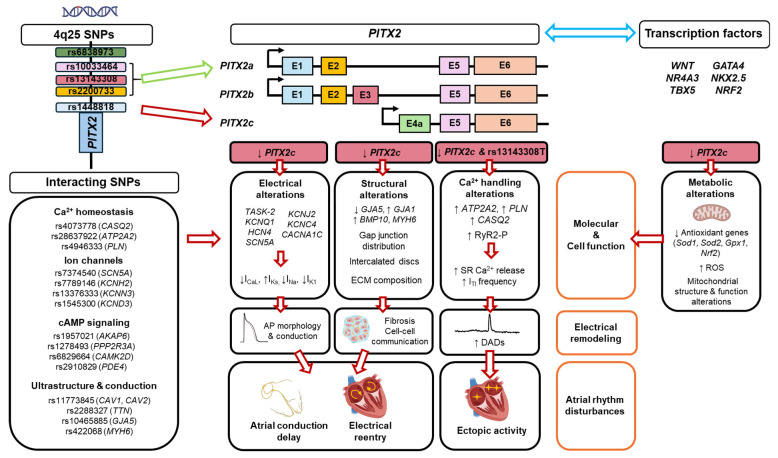
Schematic representation of the complex regulation of cell function by 4q25 SNPs and *PITX2* that leads to electrical remodeling and atrial rhythm disturbances. The figure illustrates how *PITX2c* deficiency, reported to occur for some 4q25 variants (red arrow), causes multiple alterations in molecular and cellular function that lead to both structural and electrical remodeling, known to produce atrial rhythm disturbances observed in AF. However, alterations may be potentiated by synergic interactions with other risk SNPs or transcription factors, leading to an increased net impact of the 4q25 SNPs, or be counteracted by antagonistic interactions with other SNPs or transcription factors, resulting in a reduced net impact of the 4q25 SNPs. Similarly, upregulation of *PITX2a* by other 4q25 SNPs could modify the combined impact of multiple SNPs, but this remains to be tested. E1-E6: exon 1—exon 6; I_CaL_,L-type calcium current; I_Ks_, slow rectifier potassium current; I_Na_, sodium current; I_K1_, inward rectifier potassium current; ECM, extracellular matrix; I_TI_, transient inward current; DADs: delayed afterdepolarizations; ROS, reactive oxygen species; AP, action potential.

A deeper understanding of how isoform-specific alterations in *PITX2* activity cause downstream functional disturbances is also needed. This includes dysregulation of ion channels, abnormal calcium handling, structural remodeling as well as perturbations in microRNA networks, Wnt, TGF-β and oxidative stress signaling. Furthermore, given that *PITX2* modulates key calcium and electrophysiological pathways, it will be important to define genetic interactions between 4q25 SNPs and other SNPs affecting calcium homeostasis or ion channel function, cAMP signaling and conduction of the electrical signal (see Figure 3). Identifying combinatorial SNP effects with synergistic or deleterious impact on atrial electrophysiology will support better mechanism-based polygenic risk models. To achieve this, integrated functional genomics, proteomics, multi-omic perturbation screens, and high-throughput experimental models will be needed to capture the complete *PITX2*-related effects.

In particular, the identification of SNPs that work in concert with 4q25 SNPs, enhancing their deleterious is necessary to improve the predictive effect sizes, which are currently modest. This will also afford personalized mechanism-based therapy as opposed to current polygenic risk scores that primarily allow for identifying patients with the highest (top-20%) SNP-burden, which is expected to increase AF risk by approximately 10% independently of the continuous discovery of new risk variants [117]. Similarly, the identification of SNPs that antagonize functional derangements induced by 4q25 risk SNPs may afford new insights into new molecular targets. However, larger, multi-ethnic cohorts and randomized trials will be required to test genetics-informed risk prediction and validate personalized therapies.

Future studies must also explore how *PITX2*-mediated genetic susceptibility interacts with environmental [118,119] and systemic stressors such as sex and aging [97,120,121], oxidative stress [79,122,123], inflammation [79,124], pressure overload [125,126], metabolic dysfunction [79,127], sleep apnea [80,81,82], or pharmacological treatments [19,44,84,128,129,130,131,132] known to affect cardiomyocyte function and the induction of AF. This supports the idea that an additional stress or trigger is needed, which may explain why most individuals with AF-associated variants develop the disease later in life.

Thus, translating these genetic and molecular insights into clinical tools is a critical next step. Developing polygenic high-risk profiles may enhance AF prediction and enable personalized treatment strategies based on an individual’s *PITX2* and SNP profile. Equally important is the identification of novel circulating biomarkers reflecting *PITX2* dysfunction, which could predict AF recurrence, progression, or treatment response. Ultimately, targeting key components of the *PITX2* regulatory network, such as specific microRNAs, epigenetic modulators, or upstream transcriptional regulators, may facilitate the development of targeted treatments to preserve atrial function and slow AF progression. In conclusion, combining validated high-risk SNP profiles, acting synergically on specific signaling pathways, with circulating biomarkers and established clinical risk factors may ultimately provide more robust, personalized approaches to AF management and clinical application.

## Figures and Tables

**Table 1:** Summary of AF-associated SNPs at 4q25, their reported effects on *PITX2* expression or regulation, consequences for atrial function, and implications for risk stratification and therapy.

Risk SNP	Effect on PITX2 Activity	Impact on Atrial Function	Risk Stratification	Therapeutic Relevance
rs2200733	Inconsistent effects Enhancer-related	↓ refractory period ↑ PR interval	Predictor of AF recurrence after ablation/cardioversion	↑ recurrence after ablation
rs10033464	Unclear *PITX2c* effect Enhancer-related	↑ LA volume Impaired compliance	↑ AF risk	AAD response: Class I > Class III in carriers
rs17042171 (linked with rs2200733)/rs6843082 (linked with rs10033464)	↑ *PITX2a*, =*PITX2c*	Isoform-specific effects To be tested	Isoform-dependent risk To be tested	rs17042171 (↑ recurrence after CV) rs6843082 (Exploratory)
rs2595104 (linked with rs1448818)	↓ *PITX2c* Enhancer: TFAP2a	Remains to be tested	↑ AF susceptibility	Exploratory
rs13143308	Not reported	↑ Ca^2+^ release ↑ afterdepolarizations	↑ AF risk	Therapy targeting SR Ca^2+^ release

Abbreviations: AF, atrial fibrillation; *PITX2*, paired-like homeodomain transcription factor 2; LA, left atrium; AAD, antiarrhythmic drug; CV, cardioversion.

**Table 2:** Molecular, electrophysiological, structural, and metabolic alterations induced by *Pitx2c* deficiency across experimental models and their alignment with observations in patients with AF.

Alteration	Mechanism	Pitx2c Deficient Models	AF Patients
**Calcium** **handling**	I_CaL_ density	↓	↓ ^χ^
	*CACNA1C* expression	↓/↑	↓
	SR Ca^2+^ load	↑	↑/= ^#^
	SERCA2a	↑	↓
	PLN	↑	=
	CASQ2	↑	↓
	RyR2-P	↑	↑
	Ca^2+^ sparks/waves	↑	↑
	I_TI_	↑	↑
**Electrical** **remodeling**	APD	↓	↓/↑
	RMP	Slightly depolarized	Slightly depolarized
	Conduction	Slowed	Slowed
	DADs	↑	↑
	SA Node Function	Altered	Altered
**Structural** **remodeling**	Gap junctions	↓ Cx40, ↑Cx43	↓ Cx40, ↓ Cx43
	ECM	↑ Fibrosis	↑ Fibrosis & collagen
	Atrial size	Enlarged; ↑ BMP10	Enlarged
	Sarcomeric structure	Altered	Altered
**Metabolic &** **Mitochondrial** **Alterations**	ROS	↑	↑
	Mitochondrial Function & Structure	Altered	Altered
	Adipose-like tissue	↑	↑
	Glycolysis	↑	↑

Abbreviations: AF, atrial fibrillation; SR, sarcoplasmic reticulum; APD, action potential duration; RyR2, ryanodine receptor type 2; RMP, resting membrane potential; ECM, extracellular matrix; ROS, reactive oxygen species; I_CaL_, L-type calcium current; I_TI_, transient inward current; SERCA2a, sarcoplasmic reticulum Ca2+-ATPase 2a; PLN, phospholamban; CASQ2, calsequestrin 2. Cx40, connexin 40; Cx43, connexin 43. ^#^ Load increases in patients with paroxysmal AF [76], but is unchanged or reduced in permanent AF [70,96] ^χ^ Decreased in males with permanent AF, unchanged in females and in patients with paroxysmal AF [97].

## Data Availability

Not applicable, all data are available in manuscript.

## Abbreviations

The following abbreviations are used in this manuscript:

AF	Atrial fibrillation
APD	Action potential duration
GWAS	Genome Wide Association Studies
SR	Sarcoplasmic reticulum
RyR2	Ryanodine receptor 2
I_CaL_	L-type calcium current
SNPs	Single-Nucleotide Polymorphisms
I_Ks_	Slow rectifier potassium current
I_TI_	Inward transient current
